# Measuring self-care in the general adult population: development and psychometric testing of the Self-Care Inventory

**DOI:** 10.1186/s12889-022-12913-7

**Published:** 2022-03-28

**Authors:** Michela Luciani, Maddalena De Maria, Shayleigh Dickson Page, Claudio Barbaranelli, Davide Ausili, Barbara Riegel

**Affiliations:** 1 Department of Medicine and Surgery, University of Milano – Bicocca, Via Cadore 48, 20900 Monza, Italy; 2 Department of Biomedicine and Prevention, University of Rome “Tor Vergata”, Rome, Italy; 3 School of Nursing, University of Pennsylvania, Philadelphia, USA; 4 Department of Psychology, Sapienza University of Rome, Rome, Italy

**Keywords:** Self-care, Psychometrics, General adult population, Public health, Middle range theory of self-care of chronic illness

## Abstract

**Background:**

Self-care is important at all stages of life and health status to promote well-being, prevent disease, and improve health outcomes. Currently, there is a need to better conceptualize self-care in the general adult population and provide an instrument to measure self-care in this group. Therefore, the aim of this study was to develop and evaluate the Self-Care Inventory (SCI), a theory-based instrument to measure self-care in the general adult population.

**Methods:**

Based on the Middle Range Theory of Self-Care, the 20-item SCI was developed with three scales: Self-Care Maintenance (8 items), Self-Care Monitoring (6 items), and Self-Care Management (6 items). A cross sectional study with a US-based sample (*n* = 294) was conducted to test the SCI. Internal validity was assessed with Confirmatory Factor Analysis. Internal consistency reliability was assessed with Cronbach alpha for unidimensional scales or composite reliability and the global reliability index for multidimensional scales. Construct validity was investigated with Pearson correlation to test the relationship between general self-efficacy, positivity, stress, and self-care scores.

**Results:**

The Self-Care Maintenance and Management scales were multidimensional and the Self-Care Monitoring scale was unidimensional. The global reliability index for multidimensional scales was 0.85 (self-care maintenance) and 0.88 (self-care management). Cronbach alpha coefficient of the self-care monitoring scale was 0.88. Test-retest reliability was 0.81 (self-care maintenance), 0.91 (self-care monitoring), and 0.76 (self-care management). The General Self-Efficacy Scale was positively related to all three self-care scale scores: self-care maintenance *r* = 0.46, *p* < 0. 001, self-care monitoring *r* = 0.31, *p* < 0. 001, and self-care management *r* = 0.32, *p* < 0. 001. The positivity score was positively related to self-care maintenance (*r* = 0.42, *p* < 0. 001), self-care monitoring (*r* = 0.29, *p* < 0. 001), and self-care management (*r* = 0.34, *p* < 0. 001) scores. The perceived stress was positively related to the self-care management (*r* = 0.20, *p* < 0. 001) score.

**Conclusions:**

The SCI is a theoretically based instrument designed to measure self-care in the general adult population. Preliminary evidence of validity and reliability supports its use in the general adult population.

## Introduction

Ensuring healthy lives and promoting well-being at all ages is one of the World Health Organization 2030 sustainable development goals [1]. A promising strategy to advance health for all and improve health outcomes is self-care, a person-centered approach grounded in human rights and health equity [2]. However, the World Health Organization stressed the urgent need to better conceptualize self-care to support clinical practice and interventions [3].

A recent theory [4] defined self-care as a complex and dynamic process performed throughout life of maintaining health through health-promoting practices and recognizing and managing symptoms when they occur [4, 5]. Self-care is composed of three dimensions: health promotion and treatment adherence (self-care maintenance), body listening and symptom recognition (self-care monitoring), and taking action to manage signs and symptoms (self-care management) [4, 5]. This conceptualization highlights the importance of approaching self-care as a process rather than a collection of single actions or behaviors and acknowledges that several determinants, both modifiable and non-modifiable, influence self-care [4, 5].

In the last decade, international research on self-care has focused on chronic diseases [6] with the development of several instruments to measure it, either with a general illness [7, 8] or a disease specific focus [9–11]. In chronic illness, higher levels of self-care have been associated with better health outcomes [12, 13] including decreased hospitalization, costs, and mortality [14, 15]. Previous research also focused on determinants of self-care. For example, higher positivity [16] and lower perceived stress level [17] were associated with higher self-care maintenance, self-care monitoring, and self-care management during the covid-19 pandemic and in people with diabetes, respectively. Self-efficacy was theoretically identified as a core antecedent of self-care [4, 18], and in chronic illness, higher self-efficacy was associated with higher self-care [10, 16, 17, 19].

Since the process of self-care includes behaviors, activities, decision-making and problem-solving, and since the theory states self-care is performed in “both ill and healthy states” [4], we hypothesized that this middle-range theory could be successfully used as a theoretical framework to develop an instrument to measure self-care in the general adult population [20, 21], namely every adult with or without a health condition. Indeed, while much of the existing research studies self-care in people with an illness, we argue that chronic conditions (e.g., myopia) are even more common than chronic illnesses. Everyone needs to perform self-care to promote health and prevent or delay the onset of disease, regardless to their health status.

Currently, no instrument is adequate to fully recommend that it be used to measure self-care in the general population, either in practice or in research [20]. All the instruments that measure self-care in the general adult population are either not theory based or based on Orem’s Self-Care Theory, which focuses on nursing’s role in helping people perform self-care [20]. Having a valid, reliable, and theoretically grounded instrument to measure self-care in the general adult population could help to identify people and communities at risk of poor self-care and design targeted interventions to promote self-care. Furthermore, since this instrument could be administered regardless of health status or age, it could provide valuable information on how people perform self-care throughout the lifespan. This may provide insight into how self-care is impacted by life events, social variables, and changes with age and other factors. Therefore, this study aimed to: (a) develop the Self-Care Inventory (SCI), a theory-based instrument to measure self-care in the general adult population, (b) test the structural validity of the instrument in an US sample and (c) test its reliability and construct validity by exploring the association of self-care with positivity, stress, and general self-efficacy.

## Methods

### Development

The Self-Care Inventory (SCI) was developed based on the Middle Range Theory of Self-Care of Chronic Illness [4] and a previous instrument (Self-Care of Chronic Illness Inventory - SC-CII) that evaluates self-care in people with a chronic illness [8]. A nurse theorist (BR) and two doctorally prepared nurse researchers (ML, DA) adapted the SC-CII to be applicable to the general adult population, who may or may not have pre-existing conditions. We reworded some items to apply to a broader spectrum of conditions or an overall healthy population. In items asking about medications (items #6 and #10), we reworded “take medications” to “*If/when prescribed*, take medications”, in item #9 asking about “monitor your condition” we changed it to “monitor your *health status*”. We removed any reference to chronic illnesses, for example item #14 in the SC-CII states “Many patients have symptoms due to their illness or due to the treatment they are receiving for their illness. The last time you had symptoms how quickly did you recognize it as a symptom of your illness?”. In the SCI, this item was modified to: “Think about the last time you had a symptom. This can be a symptom of anything – a cold, a bad night’s sleep, an illness. It could also be a reaction to a medicine. How quickly did you recognize it as a symptom of an illness, health problem or medicine side effect?”. Lastly, we provided examples in item #5 about what we intended for routine health care for people without a chronic illness, such as “routine check-ups, dentist, gynecologist”. Following these adaptations, the questionnaire included 20 items across three scales: Self-Care Maintenance (8 items), Self-Care Monitoring (6 items) and Self-Care Management (6 items). We maintained the 5-point Likert response of previous scales [9], with 1 being “Never” or “Not Likely” and 5 being “Always” or “Very Likely”. Content validity testing was deemed unnecessary being the instrument is strongly grounded on theoretical propositions and adapted from an existing instrument with content validity. The final version of the SCI is free to use for non-commercial research purposes after completion of an Instrument Use Agreement. The full instrument and the scoring algorithm are available at: http://self-care-measures.com/.

### Testing

To test construct validity, we developed three hypothesis based on previous studies. Findings from studies of self-care measures suggest that the Self-Care Maintenance and Self-Care Management scales would have two factors while the Self-Care Monitoring scale would have one factor [8, 22] *(Hypothesis 1)*. Moreover, we expected higher positivity [16] and lower stress levels [17] would be significantly associated with higher self-care maintenance, monitoring, and management *(Hypothesis 2)*. Finally, if the SCI measures the concepts described in the theoretical framework [4, 5], higher general self-efficacy was hypothesized to be significantly associated with higher self-care maintenance, monitoring, and management *(Hypothesis 3)*.

Following ethical approval from the Institutional Review Board of the University of Pennsylvania, a US-based sample was recruited through ResearchMatch.org, a web-based electronic registry sponsored by the US National Institutes of Health where people volunteer to participate in research studies. A total of 501 invitations to participate in the study were sent out electronically through the ResearchMatch.org website. Inclusion criteria were comprehending English and being ≥18 years old. No exclusion criteria were applied. Since the instrument is developed to be applied to any health state, we did not set a criterion on presence of a health condition; people both with and without a health condition were able to participate. Participants completed the study instruments electronically using Qualtrics (Provo, UT). To measure test-retest reliability, invitations to complete the SCI again after 10 days were sent to the first 125 participants who agreed to be recontacted after completing the instruments [23].

We administered the Self-Care Inventory [24], which required an average time of 3 min (71 s – 7 min) to complete. For hypothesis testing, we administered the following instruments. The 8-item *Positivity Scale* [25] measures the tendency to view life and experiences with a positive outlook. Participants can rate every statement from 1 (strongly agree) to 5 (strongly disagree); higher scores indicate a higher level of positivity. The positivity scale has a high level of measurement invariance among gender and countries, good internal consistency reliability (Cronbach’s α = .79), construct validity [25] and convergent validity [26]. The 10-item *Perceived Stress Scale* [27] is a tool to assess stress in the general population. Participants can score 6 negative and 4 positive statements from 0 (never) to 4 (very often) with higher scores indicating a higher level of stress. Internal consistency reliability (Cronbach’s α) in a US sample was .91 [28] and construct validity has been proven high in different studies [28, 29]. The *General Self-Efficacy Scale* [30] is an 8-item measure that assesses how much people believe they can achieve their goals in life, despite difficulties. Participants can rate every statement from 1 (strongly disagree) to 5 (strongly agree); higher scores indicate higher self-efficacy. The scale has high internal consistency reliability (Cronbach’s α = .91) and high content validity [30]. Sociodemographic and clinical data were also collected.

### Statistical analysis

Sociodemographic and clinical characteristics were analyzed using descriptive statistics. As a preliminary analysis, we examined the descriptive statistics of the demographic characteristics of the sample and of the SCI items. Normality of the variables was ascertained considering both skewness and kurtosis indices.

The internal validity of the SCI was assessed with three distinct Confirmatory Factorial Analyses (CFA), one for each SCI scale. Due to the similarity of the behaviors addressed in the SCI and the SC-CII [8], we tested the factorial structure identified for the SC-CII using a confirmatory approach. Factor loadings higher than |0.30| are considered adequate [31, 32]. Item #14 regarding symptom recognition has been suggested as a distinct concept separate from self-care monitoring and self-care management. For this reason, item #14 was tested as a separate concept and excluded from the factorial analyses.

In line with the literature [33, 34], we considered the following fit indices to evaluate the appropriateness of CFA model solution: omnibus fit indices such as chi-square (χ^2^), the incremental fit indices such as the Comparative Fit Indices (CFI; values > 0.95 indicated a good fit) the Tucker–Lewis Index (TLI; values > 0.90 indicate a good fit) and the root mean square error of approximation (RMSEA; values < 0.06 indicated a good fit) [35]. Furthermore, measures of fit in the sample such as the standardized root mean square residual (SRMR; values ≤0.06 indicate a good fit) were considered as a relevant criterion. Due to the slightly skewed distribution, the Maximum Likelihood robust (MLR) method for parameter estimation was used in the CFA [36, 37].

To assess the internal consistency reliability of the SCI, Cronbach alpha was used for the unidimensional scale. The composite reliability and the global reliability index for multidimensional scales [38] coefficients were estimated for multidimensional scales [39]. In addition, the factor score determinacy was estimated for each single factor of the SCI scales. Values ≥0.7 are considered adequate [40]. The SCI test-retest reliability was tested by the intraclass correlation coefficient (ICC); a value of 0.75 demonstrates good reliability; greater than 0.90 indicates excellent reliability [23].

Finally, according to Terwee’s recommendations [41], construct validity was further investigated testing the theoretical hypotheses described above. In particular, Pearson correlation coefficient was estimated to explain the relationship between general self-efficacy, positivity, stress and self-care scores. Correlations of .10–.29 were considered as weak, .30–.49 as moderate, and  > .50 as strong [42]. Weak and moderate correlations adequately support construct validity [43]. Data were analyzed with SPSS 26.0 and Mplus 8.1 software.

## Results

### Sample and items description

Of the 294 participants (response rate 58.5%), most were female, White, with a Bachelor’s Degree, and without a chronic condition (Table 1).

**Table 1:** Sociodemographic characteristic of the sample (*N* = 294)

Variable	***N*** = 294 n (%)
**Gender**	
Female	221 (75.2)
Male	69 (23.5)
Non Binary	3 (1.0)
Prefer not to answer	1 (0.3)
**Age (*n*** **= 293)**	
19–34	95 (32.4)
35–49	62 (21.2)
50–65	67 (22.9)
> 65	69 (23.5)
**Ethnicity**^**a**^	
American Indian or Alaska Native	8 (2.7)
Asian or Asian American	14 (4.8)
Black or African American	20 (6.8)
Hispanic, Latino, or Spanish Origin	13 (4.4)
Middle Eastern or North African	4 (1.4)
White	256 (87.1)
Other	4 (1.4)
Prefer not to answer	3 (1.0)
**Education**	
Less than High School	1 (0.3)
High School Diploma or Equivalent (GED)	10 (3.4)
Some College	30 (10.2)
Associate Degree	15 (5.1)
Bachelor Degree	112 (38.1)
Master Degree	97 (33.0)
Professional Degree (JD, MD)	8 (2.7)
Doctoral Degree (PhD, EdD)	19 (6.5)
Other	2 (0.7)
**Finances**	
Have more than enough to make ends meet	159 (54.1)
Have enough to make ends meet	129 (43.9)
Do not have enough to make ends meet	6 (2.0)
**Presence of a chronic condition**	
No	171 (58.2)
Yes	123 (41.8)
**Effect of chronic condition on daily life** (*N* = 123)	
Not at all	8 (6.5)
Minimal	79 (64.2)
Moderate	30 (24.4)
A lot	6 (4.9)
**Chronic condition is** (*N* = 123)	
Treated, well under control	70 (56.9)
Treated, not quite under control	41 (33.3)
Not Treated	12 (9.8)

^a^ Multiple answers possible

Table 2 reports the descriptive statistics of the SCI items. The items with the highest score were item #8 “Do you avoid tobacco smoke?” and item #6 “If/when prescribed, take prescribed medicines without missing a dose?”. The items with the lowest score were item #19 “Call your healthcare provider for guidance?” and item #18 “Tell your healthcare provider about the symptom at the next office visit?” Regarding item #14, “how quickly did you recognize it as a symptom of an illness, health problem, or medicine side effect?”, only 1.4% of participants did not recognize the symptoms. Among participants who recognized it (98.6%), 5.1% did not recognize it quickly, 35% recognized it somewhat quickly and 26.2% recognized it very quickly.

**Table 2:** Descriptive statistics of the SCI items (*N* = 294)

Item	Mean	SD	Skewness	Kurtosis
1. Make sure to get enough sleep?	3.94	0.87	− 0.52	− 0.06
2. Try to avoid getting sick (e.g., get flu shot, wash your hands)?	4.53	0.67	−1.46	2.08
3. Do physical activity (e.g., take a brisk walk, use the stairs)?	4.09	1.02	−0.88	− 0.17
4. Eat a balanced and varied diet?	3.79	0.89	−0.48	−0.16
5. See your healthcare provider for routine health care (e.g., routine check-up, dentist, gynecologist)?	3.93	1.22	−0.95	−0.13
6. If/when prescribed, take prescribed medicines without missing a dose?	4.55	0.82	−2.11	4.51
7. Do something to relieve stress (e.g., meditation, yoga, music)?	3.50	1.19	−0.49	−0.57
8. Do you avoid tobacco smoke?	4.77	0.75	−3.72	14.07
9. Monitor your health status?	3.72	1.07	−0.55	−0.31
10. If/when prescribed, monitor for medicine side-effects?	3.91	1.13	−0.89	0.01
11. Pay attention to changes in how you feel?	4.12	0.91	−0.79	0.03
12. Monitor whether you tire more than usual doing normal activities?	3.78	1.10	−0.70	−0.17
13. Monitor for symptoms?	3.83	1.06	−0.67	−0.21
14. How quickly did you recognize it as a symptom of an illness, health problem, or medicine side effect?	3.54	1.19	−0.58	0.04
15. Change what you eat or drink to make the symptom decrease or go away?	3.45	1.37	−0.38	−1.26
16. Change your activity level (e.g., slow down, rest)?	3.36	1.35	−0.30	−1.34
17. Take a medicine to make the symptom decrease or go away?	2.99	1.39	0.01	−1.39
18. Tell your healthcare provider about the symptom at the next office visit?	2.97	1.37	0.01	−1.30
19. Call your healthcare provider for guidance?	2.52	1.17	0.20	−0.77
20. Think of things you did the last time you had a symptom–… - Did the things you did make you feel better?	3.29	1.34	−0.81	0.27

*SD* standard deviation

### Structural validity and reliability

#### Self-care maintenance

**Fig. 1:**
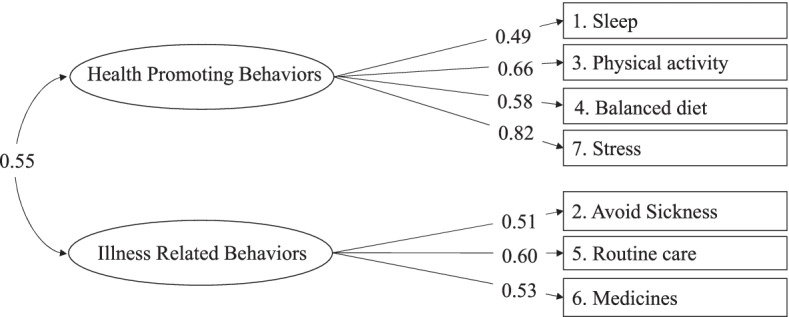
Graphic representation of the Self-Care Maintenance scale of the SCI. *Note.* Results of the confirmatory factor analysis with the full sample of 294. The standardized solution of the Mplus output is reported

In the SC-CII, Self-Care Maintenance is described as comprising “health-promoting behaviors” and “illness-related behaviors” factors, measured by four and three items (an eighth item is related to both dimensions), respectively. For this reason, we specified a two-factor confirmatory model. The goodness-of-fit indices of this model were poor. As with the SC-CII [8], item #8 (How often do you avoid tobacco smoke) had a nonsignificant factor loading. We believe that avoiding tobacco smoke is important, so we left the item in the scale, but excluded it from the analysis. In addition, item #4 produced a modification index of 32.78 in “illness-related behaviors” factor. Thus, a second CFA model with seven items and the allocation of item #4 to “illness-related behaviors” was run and the model had an excellent fit to the data: χ^2^ (13, *N* = 294) = 23.009, *p* = 0.042, CFI = 0.967, TLI = 0.947, RMSEA = 0.051 (90% CI = 0.010 0.085), *p* = 0.435, SRMR = 0.043. Thus, the items from #1 to #4 loaded on the health-promoting behaviors factor while items from #5 to #7 loaded on the illness-related behaviors factor. The two factors were positively and significantly correlated (*r* = 0.55, *p* < 0.001). All factor loadings were adequate and > 0.49 **(**Fig. 1**)**.

Due to the multidimensional structure of the Self-Care Maintenance scale, the composite reliability coefficient was 0.73 and the global reliability index for multidimensional scales was 0.85. The factor score determinacy coefficient was 0.89 and 0.79 for the health promoting behaviors and illness related behaviors factor, respectively. The test-retest reliability was 0.81 for this scale.

#### Self-care monitoring

**Fig. 2:**
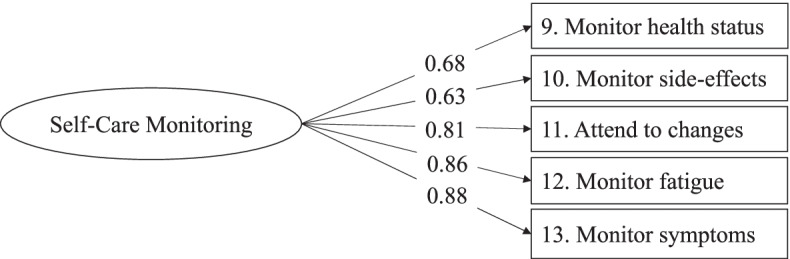
Graphic representation of the Self-Care Monitoring scale of the SCI. *Note.* Results of the confirmatory factor analysis with the full sample of 294. The standardized solution of the Mplus output is reported

The Self-Care Monitoring scale is described as comprising five items, from item #9 to #13. Thus, we specified an one-factor model CFA that yielded an excellent fit: χ^2^ (5, *N* = 295) = 11.145, *p* = 0.049, CFI = 0.984, TLI = 0.868, RMSEA = 0.065 (90% CI = 0.005 0.116), *p* = 0.265, SRMR = 0.029. All items had high factor loadings, > 0.63 **(**Fig. 2**)**.

The Cronbach alpha coefficient of the Self-Care Monitoring scale was 0.88, the composite reliability coefficient was 0.86, and the factor score was 0.95. Test-retest reliability was 0.91.

#### Self-care management

**Fig. 3:**
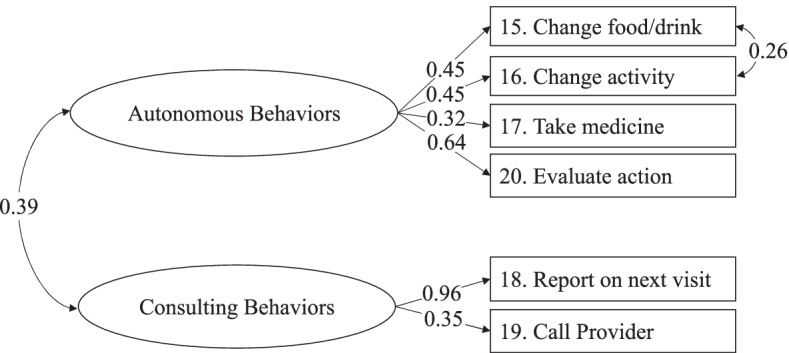
Graphic representation of the Self-Care Management scale of the SCI. *Note.* Results of the confirmatory factor analysis with the full sample of 294. The standardized solution of the Mplus output is reported

The Self-Care Management scale is described by the two dimensions of “autonomous behaviors” and “consulting behaviors”, measured by four and two items, respectively. Thus, we specified a two-factor model CFA. We hypothesized that items #15, #16, #17 and #20 were loading the autonomous behaviors factor, and items #18 and #19 the consulting behaviors factor. This posited model showed an adequate fit; the modification indices highlight that the cause of the model misfit was in the residual covariances between items #15 (Change what you eat or drink to make the symptom decrease or go away?) and #16 (Change your activity level?). Since the proximity of these two items in the instrument could increase the meaning and consequently the shared variance, we had methodological and theoretical reason to specify a post-hoc error covariance between these two items. When we re-ran the model allowing the residuals of these two items to be correlated [44, 45], the fit was excellent: χ^2^ (7, *N* = 294) = 12.093, *p* = 0.0975, CFI = 0.967, TLI = 0.929, RMSEA = 0.050 (90% CI = 0.000 0.096), *p* = 0.444, SRMR = 0.030. All factor loadings were adequate and ranged from 0.32 to 0.96, and two factors were positively and significantly correlated (*r* = 0.39, *p* < 0.001) **(**Fig. 3**)**.

The composite reliability coefficient was 0.72 and the global reliability index for multidimensional scales was 0.88. The factor score determinacy coefficients were 0.76 and 0.97 for the autonomous behaviors and consulting behaviors factor, respectively. The test-retest reliability was 0.76.

### Simultaneous confirmatory analysis

As a final step, we conducted a simultaneous CFA on the combined set of items to demonstrate that the factors underlying the scales emerge clearly, not only when each scale was analyzed separately but also when the analysis was performed on the combination of items. CFA supported this more general model with the following fit indices: χ^2^ (125, *N* = 294) = 181.822, *p* < 0.001, CFI = 0.957, TLI = 0.947, RMSEA = 0.039 (90% CI = 0.026 0.051), *p* = 0.927, SRMR = 0.048. Factor loadings and factor correlations from this solution are shown in Table 3**.**

**Table 3:** Factor loadings and factor correlation from the simultaneous confirmatory factor analysis on the full sample

**Self-Care Maintenance Scale**				**Factor Loading**
**Health-promoting behaviors**				
* How often or routinely do you…*				
1. Make sure to get enough sleep?				0.50
3. Do physical activity (e.g., take a brisk walk, use the stairs)?				0.65
4. Eat a balanced and varied diet?				0.81
7. Do something to relieve stress (e.g., meditation, yoga, music)?				0.59
**Illness-related behaviors**				
2. Try to avoid getting sick (e.g., flu shot, wash your hands)?				0.54
5. See your healthcare provider for routine health care (e.g., routine check-ups, dentist, gynecologist)?				0.60
6. If/when prescribed, take prescribed medicines without missing a dose?				0.50
**Self-Care Monitoring Scale**				**Factor Loading**
* How often or routinely do you…*				
9. Monitor your health status?				0.70
10. If/when prescribed, monitor for medicine side-effects?				0.63
11. Pay attention to changes in how you feel?				0.82
12. Monitor whether you tire more than usual doing normal activities?				0.85
13. Monitor for symptoms?				0.87
**Self-Care Management Scale**				**Factor Loading**
**Autonomous behaviors**				
* When you have symptoms, how likely are you to…*				
15. Change what you eat or drink to make the symptom decrease or go away?				0.55
16. Change your activity level (e.g., slow down, rest)?				0.53
17. Take a medicine to make the symptom decrease or go away?				0.28
20. Did the things you did make you feel better?				0.56
**Consulting behaviors**				
18. Tell your healthcare provider about the symptom at the next office visit?				0.68
19. Call your healthcare provider for guidance?				0.48
**Factors**	Illness related behaviors	Self-Care monitoring	Autonomous behaviors	Consulting behaviors
Health promoting behaviors	0.56	0.45	0.41	0.15
Illness related behaviors		0.43	0.31	0.37
Self-Care monitoring			0.69	0.29
Autonomous behaviors				0.47

Results come from Mplus completely standardized solutions. All coefficients are statistically significant (*p* < 0.05)

### Construct validity

The correlation coefficients of general self-efficacy in relation to the Self-Care Maintenance scale, the Self-Care Monitoring scale, and the Self-Care Management scale were 0.42, 0.35 and 0.35, respectively. The General Self-Efficacy Scale was positively and moderately related to all three self-care scale scores: self-care maintenance *r* = 0.46, *p* < 0. 001, self-care monitoring *r* = 0.31, *p* < 0. 001, and self-care management *r* = 0.32, *p* < 0. 001. The positivity score was positively and moderately related to self-care maintenance (*r* = 0.42, *p* < 0. 001), weakly related to self-care monitoring (*r* = 0.29, *p* < 0. 001) and moderately related to self-care management (*r* = 0.34, *p* < 0. 001) scores. The perceived stress was positively and weakly related to the self-care management (*r* = 0.20, *p* < 0. 001) score.

### Self-care inventory scales scores

Each of the SCI scale scores were calculated. The mean scores were 76.16 (±14.70), 71.77 (±21.66) and 52.38 (±19.06) for self-care maintenance, self-care monitoring and self-care management, respectively.

## Discussion

The aim of this study was to develop and test a theory-based instrument, the Self-Care Inventory (SCI), to measure self-care in the general adult population. We adapted an existing theory-based [4] self-report instrument measuring self-care in individuals with chronic illness [8] to be generic and useful in the general adult population. The initial psychometric testing shows that the instrument can be used in people with or without a health condition. We found adequate reliability and construct validity. An instrument able to measure self-care in the general adult population could be valuable to large scale administration and longitudinal studies, and useful in comparing self-care across populations and interventions in public health studies.

Other instruments based on this theory use a cut-point of ≥70 to indicate adequacy of self-care [46]. Similar to other populations [47, 48], in this sample we saw adequate self-care maintenance and self-care monitoring behaviors. Yet, the item mean scores were notably lower in the Self-Care Management scale, especially in items inquiring about calling a healthcare provider for guidance, telling the provider about the symptom at the next office visit, and taking a medicine to make the symptom decrease or go away. It may be that this generally healthy population has not established a relationship with a healthcare provider. In the United States, the proportion of adults who have an identified primary care provider has declined in recent decades [49]. This trend is particularly pronounced among adults who report no comorbidities, with only 51% reporting a primary care provider as of 2015 [49]. This trend may be explained by financial barriers to care that are especially pronounced among the uninsured or under-insured [50], access barriers related to primary care physicians shortages [51, 52], or changing models of healthcare delivery that focus on convenience rather than continuity of care (e.g., urgent care clinics located within retail stores, telehealth consults) [53]. Another possible explanation for lower scores on the Self-Care Management scale is that individuals are consulting internet sources of health information, rather than a healthcare provider, to obtain information about a condition or guidance on how to respond to the symptom [54–56]. Finally, this population may eschew medications at this stage of life. Probably this is why item #17 showed a lower factor loading compared with the other items of the scale. However, the conjoint solution was excellent, allowing us to consider the Self-Care Management scale as reliable. Finally, the factorial structure of the three scales were confirmed by the CFA and coherent with those of previous self-care instruments [8, 22].

In terms of validity testing, we hypothesized that higher general self-efficacy, higher positivity, and lower stress levels would be associated with higher self-care. We found that higher general self-efficacy was significantly associated with higher scores in all dimensions of self-care. In other words, individuals who felt confident in their ability to achieve their goals in life were more likely to engage in health-promoting behaviors, monitor their health for changes, and take action when a symptom occurs. This is consistent with the theoretical framework [4] and lends support to the construct validity of the SCI.

As hypothesized, higher positivity was associated with all dimensions of self-care. This suggests that individuals with a more positive outlook on life engage in more self-care behaviors. The strongest association was found between positivity and self-care maintenance, which reflects behaviors that individuals do proactively to promote health and avoid illness. This finding is consistent with previous research that shows that positivity is associated with health promoting behaviors, such as quitting smoking [57] and eating fruits and vegetables [58].

It was surprising that higher perceived stress was associated with higher self-care management, because prior investigators have shown that higher stress is related to worse self-care [59]. Psychological stress has deleterious effects on multiple body systems. For example, stress is associated with immune dysregulation, which makes one more susceptible to infectious diseases [60], impaired functioning of the gastrointestinal tract [61], and at risk for cardiovascular disease [62]. Thus, it is possible that individuals with higher perceived stress are more likely to experience symptoms and therefore engage more in self-care management behaviors to treat these symptoms. Higher stress levels may also reflect worry, which could generate more attention to symptoms and self-care.

### Strengths and limitations

A few limitations should be acknowledged. We used a convenience sample that was predominantly female, White, and well educated. This reflects the demographics of volunteers enrolled in Researchmatch.org, but further research is needed to ensure that the instrument is reliable and valid in diverse populations and those in other countries. A study is underway now to validate the SCI in Italy and in a longitudinal study of caregivers in the US [63]. Strengths include a good response rate for an online survey [64] and a varied distribution between people with and without previous conditions.

## Conclusion

The Self-Care Inventory (SCI) is theory-based instrument to measure self-care in the general adult population, preliminary evidence of validity and reliability supports its use and further testing in this population. The SCI was adapted from the Self Care of Chronic Illness Inventory (SC-CII) to be administered regardless of health status. In this sample of US adults, reliability was adequate across all scales and there was strong evidence for construct validity.

There are valuable potential applications for this instrument. The SCI can be used in research to better understand self-care in adults regardless of age or presence of a chronic condition. The process of self-care in its three dimensions of self-care maintenance, self-care monitoring, and self-care management has not been described before in the general population. In clinical practice, the SCI can help to identify people, caregivers, or populations who are at risk for decreased self-care and, hence, poor health outcomes. Future studies will need to test the validity of the instrument in different and diverse samples, and possibly verify the construct validity longitudinally.

## Data Availability

The datasets used and analyzed during the current study is available from the corresponding author on reasonable request.
